# Winsley Sanatorium

**Published:** 1921-02-19

**Authors:** 


### Winsley Sanatorium.

At the half-yearly meeting of the Governors of Winsley Sanatorium held at the institution recently, the Executive Committee presented a very full report. In view of the increasing number of applications for admission, and the knowledge that all beds have been continuously occupied during the past year, the large extension contemplated in the near future is greatly needed. It is not very clear from the report whether the money to be provided by a certain lady for the benefit of poor consumptives in Winsley is at present available. The resignation of the Medical Superintendent was announced. A house is to be built shortly for his successor, suitable accommodation for whom is very pressing. It seems a pity in these days of house shortage to have had to convert "The Cottages" into quarters for nurses, but presumably that was the only alternative. It was considered advisable to refuse the application from the Ministry of Health to set up a training centre for forty ex-Service men, and also the offer from the Wilts County Council to purchase part of the sanatorium estate. It would certainly seem a wise provision not to prejudice the future efficient working of the institution by any present compromise. Winsley Sanatorium commands support from many different public bodies in the neighbourhood, and it is not surprising to learn that more accommodation is now needed.

